# Supplementary figures and images for: Wastewater surveillance in smaller college communities may aid future public health initiatives
Source: PLoS One. 2022 Sep 16;17(9):e0270385. doi: 10.1371/journal.pone.0270385 (PMC9481015; doi:10.1371/journal.pone.0270385)

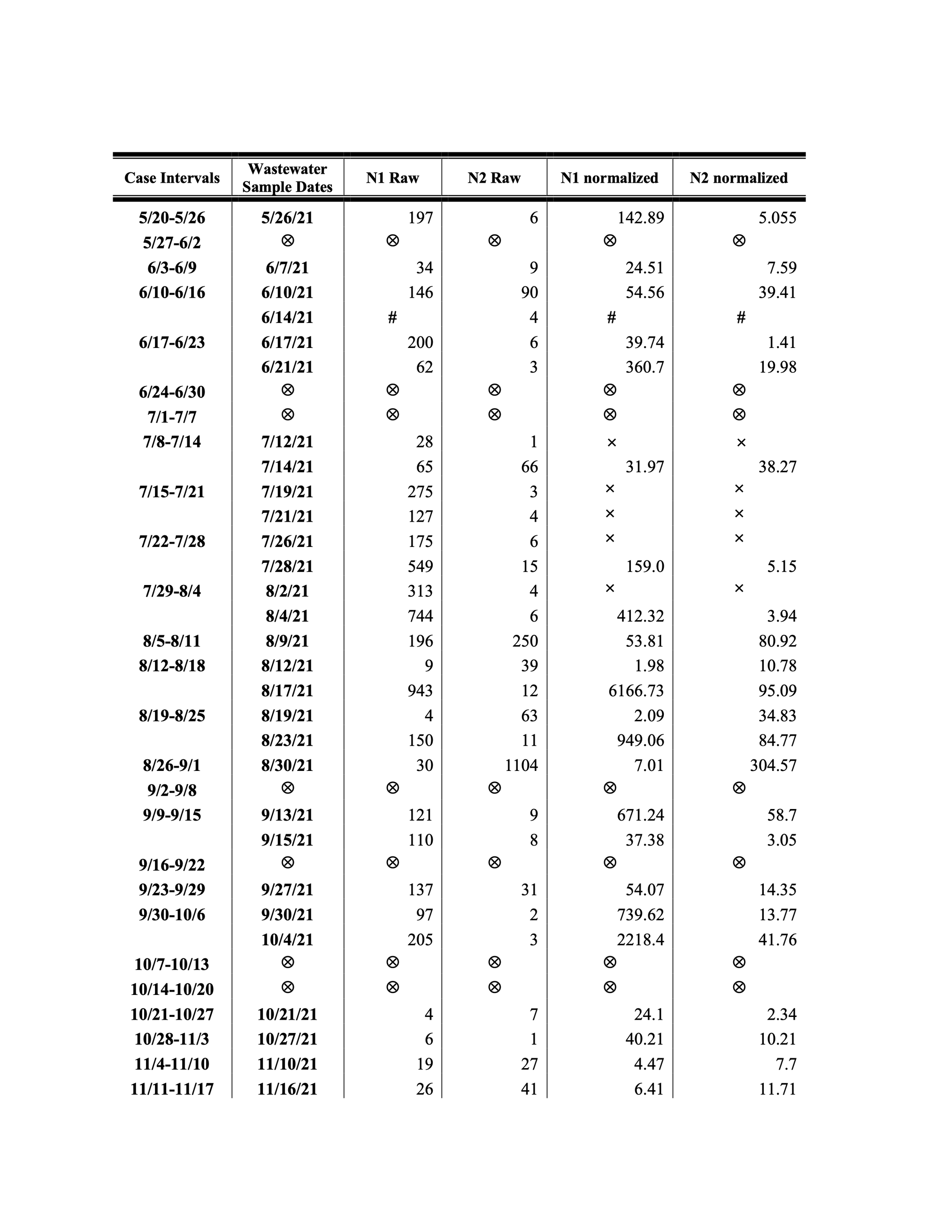

Supplement: S1 Table — (TIF) [file pone.0270385.s001.tif]
